# Supplementary material for: Characterization and Modification of Light-Sensitive Phosphodiesterases from Choanoflagellates
Source: Biomolecules. 2022 Jan 6;12(1):88. doi: 10.3390/biom12010088 (PMC8774190; doi:10.3390/biom12010088)
Supplement: Supplementary file 1 [file biomolecules-12-00088-s001.zip › biomolecules-1495314-supplementary.pdf]

biomolecules

MDPI

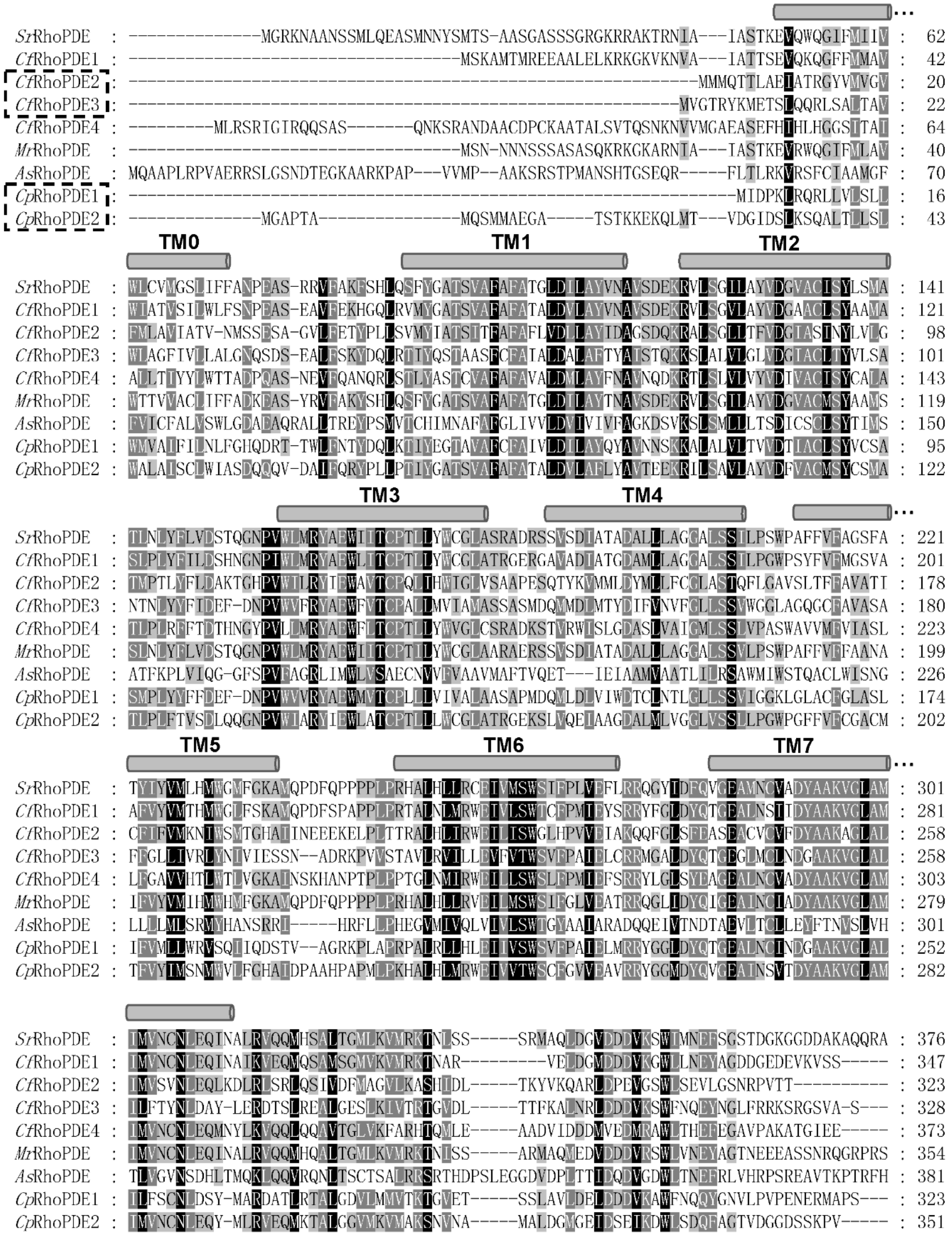

Figure S1. The alignment of full rhodopsin domains of all the RhoPDEs. The predicted transmembrane helices were labeled from TM0 to TM7. *Cf*RhoPDE2, 3 and *Cp*RhoPDE1, 2 have no cGMP hydrolysis activity with shorter N-terminal fragments. The deeper background color, the more conserved residues in the sequences.
